# Anatomical Location of the Vestibulocerebellar Tract in the Healthy Human Brain: A Diffusion Tensor Imaging Study

**DOI:** 10.3390/brainsci11020199

**Published:** 2021-02-05

**Authors:** Seo Yoon Park, Sang Seok Yeo, Sung Ho Jang, In Hee Cho

**Affiliations:** 1 Department of Health, Graduate School, Dankook University, Cheonan-si 31116, Korea; pgy0614@hanmail.net; 2 Department of Physical Therapy, College of Health Sciences, Dankook University, Cheonan-si 31116, Korea; eangbul@hanmail.net; 3 Department of Physical Medicine and Rehabilitation, College of Medicine, Yeungnam University, Gyeongsan-si 42415, Korea; strokerehab@hanmail.net

**Keywords:** vestibulocerebellar tract, diffusion tensor imaging tractography, visual vertical, vestibular system

## Abstract

The vestibulocerebellar tract (VCT) is regarded as an important pathway of the central vestibular system. We identified the anatomical characteristics of the primary and secondary VCTs in a normal human brain using diffusion tensor imaging (DTI) tractography. Thirty-one healthy adults were recruited. A 1.5 T scanner was used for DTI tractography. A seed region of interest (ROI) was placed on the superior and medial vestibular nuclei at the pons level and a target ROI was placed on the uvula–nodulus of the cerebellum for reconstructing the primary VCT. In the secondary VCTs, the seed ROI was placed on the inferior and medial vestibular nuclei at the medulla oblongata level, and target ROIs were placed on the bilateral uvula–nodulus of the cerebellum. The primary VCT originated from the superior and medial vestibular nuclei at the pons level and terminated at the ipsilateral uvula–nodulus of the cerebellum. The component of the secondary VCTs originated from the inferior and medial vestibular nuclei at the level of the medulla oblongata and terminated at the bilateral uvula–nodulus of the cerebellum. Among them, 70.97% in the contralateral secondary VCT crossed at the vermis of the cerebellum. In addition, the fractional anisotropies (FAs) and mean diffusivity (MD) values of the primary VCT were significantly higher and lower, respectively, compared to those of the secondary VCTs (*p* < 0.05). The contralateral secondary VCT was significantly higher and lower in the MD and tract volume, respectively (*p* < 0.05), compared to the ipsilateral VCT. Therefore, we believe that the results will be useful for future studies of the vestibular projection pathway in the human brain injury aspect of central vestibular syndrome.

## 1. Introduction

The vestibular system is composed of the peripheral vestibular organs in the inner ear, the ocular system, and projections of the central nervous system [1,2]. Particularly, the vestibular system in the central nervous system is controlled by the interaction of the cerebrum, brainstem, and cerebellum. Among them, the cerebellum is functionally divided into three branches: the vestibulocerebellum, spinocerebellum, and corticocerebellum [3,4,5,6]. These three branches involve tracts that control the functions for which these branches are responsible: the vestibulocerebellar (VCT), spinocerebellar, and corticocerebellar tracts [3,4,5]. The VCT is regarded as an important pathway of the central vestibular system and is known to play a vital role in the control of eye movements and postural reflexes, as well as the perception of head motion and spatial orientation [1,2,4,5,7]. Therefore, a few studies have investigated the integrity of the VCT by assessing vestibular function [3,5]

Previous studies have suggested that the VCT is divided into primary and secondary projections and reported that primary and secondary VCTs connect the vestibular nucleus with the uvula–nodulus of the cerebellum [1,4,8,9,10]. The primary and secondary vestibular mossy fibers are projected from the vestibular nuclei to the ipsilateral and bilateral uvula–nodulus of the cerebellum, respectively [8,9,11]. Although a secondary VCT is known to connect the inferior and medial vestibular nucleus to the bilateral uvula of the cerebellum, this has only been studied in the animal brain, and it is not clear where the contralateral secondary VCT crosses [1,4,8,9,10,12]. Diffusion tensor imaging (DTI) tractography quantifies diffusion in three-dimensional directions and enables the visualization of anatomical structures by imaging water diffusion patterns [13,14,15] Previous studies have investigated the function of the VCT and reconstructed the VCT in the brains of animals [6,8,11] In addition, one study by Jang et al. reconstructed the primary VCT in the normal human brain using DTI tractography [10]. As a result, they identified that the primary VCT was projected from the superior and medial vestibular nuclei and passed inferoposteriorly before being terminated at the uvula–nodulus of the cerebellum.10 As of yet, no studies have reconstructed the secondary VCTs in the normal human brain using DTI tractography.

In the current study, we reconstructed the structures and identified the anatomical characteristics of the primary and secondary VCTs in the normal human brain using DTI tractography.

## 2. Material and Methods

### 2.1. Subjects

This study recruited thirty-one healthy adults (aged 20–40 years) that did not have any history of neurological or musculoskeletal disease. The inclusion criteria were: (1) no diagnosis associated with vestibular function in the past, (2) no history of cerebellum injury, and (3) no history of neurological, cognitive, or musculoskeletal dysfunction. The exclusion criteria were: (1) participants who had been diagnosed with musculoskeletal and neurological problems in the past and (2) participants who had been diagnosed with a brain injury in the past. All participants provided informed consent prior to DTI tractography. The study was approved by the institutional review board of Dankook University (DKU 2020-07-009).

### 2.2. Diffusion Tensor Image Tractography

The DTI data were acquired using a six-channel head coil on a 1.5 T Philips Gyroscan Intera (Philips, Best, The Netherlands) with single-shot echo-planar imaging. For each of the 32 non-collinear diffusion sensitizing gradients, this study collected 67 contiguous slices parallel to the anterior commissure–posterior commissure line. The imaging parameters used were: acquisition matrix = 96 × 96; reconstructed matrix = 192 × 192; field of view = 240 × 240 mm2; repetition time (TR) = 10,726 ms; echo time (TE) = 76 ms; parallel imaging reduction factor (SENSE factor) = 2; EPI factor = 49; b = 1000 s/mm2; NEX = 1; and a slice thickness of 2.5 mm with no gap (acquired voxel size: 1.3 × 1.3 × 2.5 mm3) [16].

### 2.3. Probabilistic Fiber Tracking

**Figure 1:**
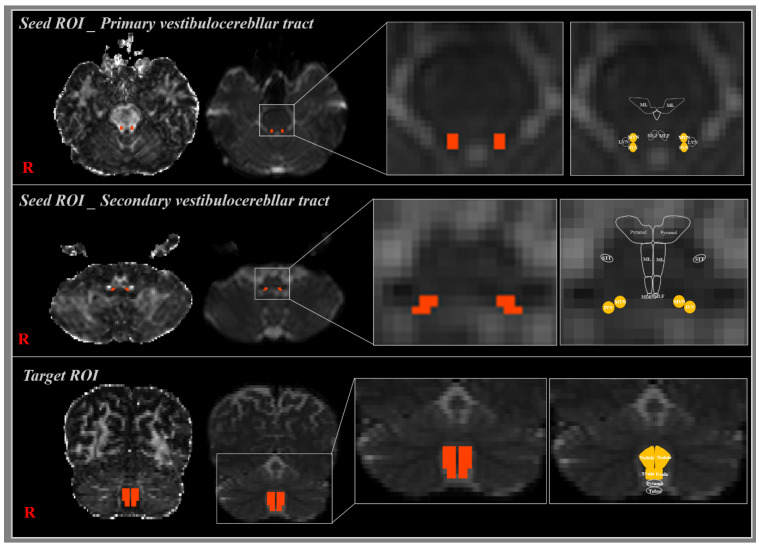
The seed region of interest (ROI) of the primary vestibulocerebellar tract (VCT) was placed on the superior and medial vestibular nuclei at the pons level. The target ROI was located in the uvula–nodulus of the cerebellum (orange rectangle). In the case of bilateral secondary VCT, the seed ROI was located in the inferior and medial vestibular nuclei at the medulla oblongata level, and target ROIs were located in the bilateral uvula–nodulus of the cerebellum (orange rectangle).

The diffusion-weighted imaging data were analyzed using the Oxford Center for Functional Magnetic Resonance Imaging of the Brain (FMRIB) Software Library (FSL; www.fmrib.ox.ac.uk/fsl). The head motion effect and image distortions due to eddy currents were corrected by affine multi-scale two-dimensional registration. Fiber tracking was performed using a probabilistic image method based on a multifiber model and by utilizing image routines implemented in FMRIB diffusion (5000 streamline samples, 0.5 mm step length, curvature threshold = 0.2) [17]. The primary and secondary VCTs were determined by selecting fibers passing through a seed region of interest (ROI) and a single target ROI. The seed and target ROIs of the ipsilateral primary VCT were located as follows: A seed ROI was located in the superior and medial vestibular nuclei at the pons level, 10 and a target ROI was located on the uvula–nodulus of the cerebellum (Figure 1) [10]. In the secondary VCTs, the seed ROI was located in the inferior and medial vestibular nuclei at the medulla oblongata level, and the target ROIs were placed on the bilateral uvula–nodulus of the cerebellum (Figure 1) [4,8]. A total of 5000 samples were generated from a seed voxel, and this study visualized the results at a minimum of one streamline through each voxel. Fractional anisotropies (FAs), mean diffusivity (MD), and tract volumes of the primary, ipsilateral, and contralateral secondary VCTs were acquired.

### 2.4. Statistical Analysis

This study analyzed the significance of differences in the DTI parameters between the three reconstructed VCTs using a one-way analysis of variance (ANOVA) with an Fisher’s least significant difference (LSD) post-hoc test. SPSS ver. 20.0 (SPSS, Chicago, IL, USA) was used for the analysis. A *p*-value of < 0.05 was considered to be statistically significant.

## 3. Results

**Figure 2:**
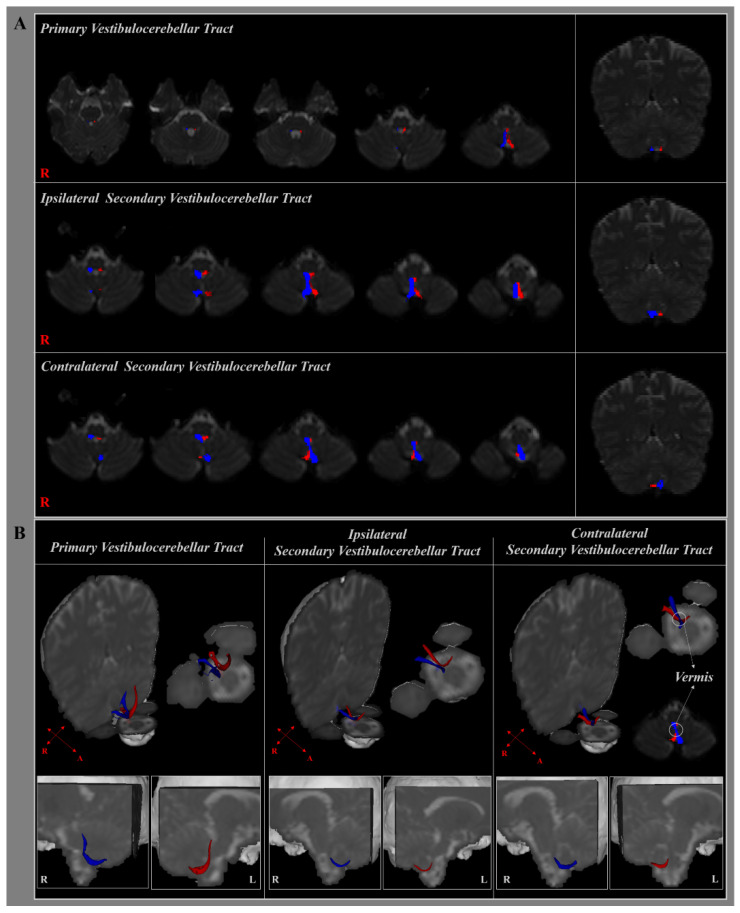
(**A**) The primary and bilateral secondary vestibulocerebellar tracts (VCTs) are shown at each level of the brainstem in a normal healthy adult according to the type of diffusion tensor imaging (DTI) tractography. (**B**) The right (blue) and left VCTs (red) are shown in three-dimensional planes in a normal healthy adult, and the cross-section of the contralateral secondary VCT is shown using DTI tractography.

Using the probabilistic DTI tractography, this study reconstructed the primary and secondary VCTs in the normal human brain (Figure 2). The primary VCT originated from the superior and medial vestibular nuclei at the pons level, passed through the inferior cerebellar peduncle, and terminated at the ipsilateral uvula–nodulus of the cerebellum (Figure 2). The component of the secondary VCTs originated from the inferior and medial vestibular nuclei at the level of the medulla oblongata, crossed at the vermis of the cerebellum on the contralateral side, and terminated at the bilateral uvula–nodulus of the cerebellum (Figure 2). In the contralateral secondary VCT, 70.97% crossed at the vermis of the cerebellum.

**Figure 3:**
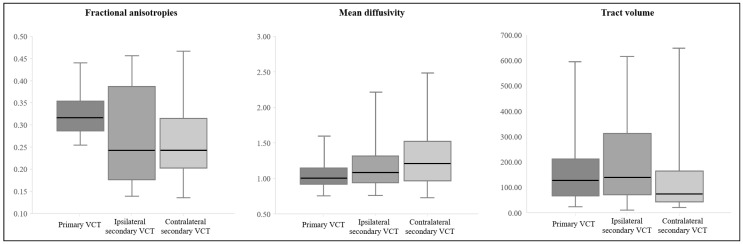
Boxplot of the diffusion tensor imaging parameters related to the vestibulocerebellar tract.

The mean FAs, MD, and tract volumes of the primary, ipsilateral, and contralateral VCTs are shown in Table 1 (Figure 2B and Figure 3). The FAs and MD parameters of the primary VCT were significantly higher and lower, respectively, compared to those of the secondary VCTs (*p* < 0.05). However, there were no significant differences in the tract volume between the primary and secondary VCTs (*p* > 0.05). The contralateral secondary VCT was significantly higher and lower in the MD and tract volume, respectively (*p* < 0.05), compared to the ipsilateral VCT. In contrast, the FAs of the DTI parameters were not significantly different between the secondary VCTs (*p* > 0.05).

## 4. Discussion

In the current study, we reconstructed the primary and secondary VCTs in the healthy human brain using DTI tractography. With our reconstruction of the VCTs, we found that: (1) The primary VCT originated from the pons level, passed through the inferior cerebellar peduncle, and terminated at the ipsilateral uvula–nodulus of the cerebellum; (2) the secondary VCTs originated from the medulla oblongata, crossed at the vermis of the cerebellum on the contralateral side, and terminated at the bilateral uvula–nodulus of the cerebellum. Regarding the FA and MD values of the primary VCT, they were significantly higher and lower, respectively, compared to those of the ipsilateral and contralateral secondary VCTs. The MD value of the contralateral secondary VCT was significantly higher than that of the ipsilateral secondary VCT. In contrast, the tract volume was not significantly different between the primary and secondary VCTs.

Previous animal studies have shown that the brain anatomy of the primary VCT terminates at the ipsilateral uvula–nodulus of the cerebellum in cats, rabbits, monkeys, and pigeons [18,19,20,21,22]. In 1993, Barmack et al. suggested that the primary VCT from the pontine medial vestibular nuclei to the cerebellum of rabbits was identified as the uvula–nodulus of the cerebellum [19]. Schwarz et al. (1983) reported that the primary VCT of pigeons was concentrated in the nodulus and the most posterior gyrus of the uvula [20]. Consequently, the anatomical pathway of the reconstructed primary VCT in this study is in agreement with the findings of these previous studies. Several histological studies using projection have reported the anatomy of the secondary VCTs in animal brains, such as galago and rabbit [11,23]. In 1981, Rubertonr et al. demonstrated that the secondary VCTs terminated on the bilateral flocculonodular in the galago brain [23]. In the rabbit brain, Thunnissen et al. (1989) suggested that the secondary VCTs from the vestibular nuclei to the cerebellum were terminated on the bilateral caudal vermis in the cerebellum [11]. In contrast, our study found that the secondary VCTs crossed the vermis of the cerebellum on the contralateral side and terminated at the bilateral uvula–nodulus of the cerebellum.

In the human brain, it is known that the primary VCT originates from the inferior cerebellar peduncle and terminates in the vestibulocerebellum of the ipsilateral side. It is also known that the secondary VCTs originate from the medial and inferior vestibular nuclei and terminate in the vestibulocerebellum (uvula, nodule, and flocculus) [24]. Our findings were consistent with the literature regarding the anatomical pathways to the VCTs. Although several studies have reported that the VCT are divided into primary and secondary VCTs [9,25], only one study investigated the anatomy of the primary VCT, and none have reconstructed the secondary VCTs in the human brain using DTI. In 2018, using DTI, Jang et al. reconstructed the primary VCT in the normal human brain. The primary VCT projected from the pons level, passed inferoposteriorly via the inferior cerebellar peduncle, and then terminated at the portion of the uvula of the cerebellum [10]. These results seem to be in agreement with the results of the current study. However, there have been no studies regarding the reconstruction and estimation of the secondary VCTs in the human brain.

In conclusion, based on the DTI tractography findings, we identified the anatomical characteristics of the primary and secondary VCTs in the human brain. We believe that the results of the current study will be clinically helpful in evaluating postural control impairment and central vestibular syndrome according to injuries of the vestibular projection pathway in patients with brain injuries, and particularly lesions of the VCT. However, some limitations of the study should be considered. First, the generalizability of our findings is difficult due to the limited age range of the subjects recruited (20–40 years), and thus, additional research needs to be performed in a population with a greater age range in order to generate standard data. Second, DTI may have difficulty in locating ROIs accurately due to the fiber tracts in regions of crossing and fiber complexity. Although DTI is a powerful anatomical imaging tool that can demonstrate a general fiber architecture, it can underestimate or overestimate all of the fibers. Therefore, we suggest that further studies be undertaken to demonstrate the reliability and validity of the VCT and of its clinical correlations.

## Figures and Tables

**Table 1:** Diffusion tensor imaging parameters of the vestibulocerebellar tract.

		FA	MD	Tract Volume
Primary VCT		0.28	1.05	176.53
		(0.06)	(0.19)	(142.50)
Secondary VCT	Ipsilateral	0.22	1.18	204.92
		(0.07)	(0.32)	(166.10)
	Contralateral	0.22	1.29	125.87
		(0.08)	(0.40)	(121.62)
A vs. B		0.000 *	0.024 *	0.276
A vs. C		0.000 *	0.000 *	0.053
B vs. C		0.902	0.043 *	0.003 *

Values represent mean (± standard deviation). * Post-hoc values show the *p*-value (*p* < 0.05). VCT, vestibulocerebellar tract; FA, fractional anisotropy; MD, mean diffusivity. A, primary VCT; B, ipsilateral secondary VCT; C, contralateral secondary VCT.

## Data Availability

The data presented in this study are available on reasonable request from the corresponding author.
